# Transitive Inference Remains Despite Overtraining on Premise Pair C+D-

**DOI:** 10.3389/fpsyg.2018.01791

**Published:** 2018-10-02

**Authors:** Héctor O. Camarena, Oscar García-Leal, José E. Burgos, Felipe Parrado, Laurent Ávila-Chauvet

**Affiliations:** Center for Studies and Investigations in Behavior, University of Guadalajara, Guadalajara, Mexico

**Keywords:** transitive inference, value transfer, reinforcement, overtraining, bias reversal

## Abstract

Transitive inference (TI) has been studied in humans and several animals such as rats, pigeons and fishes. Using different methods for training premises it has been shown that a non-trained relation between stimuli can be stablished, so that if A > B > C > D > E, then B > D. Despite the widely reported cases of TI, the specific mechanisms underlying this phenomenon remain under discussion. In the present experiment pigeons were trained in a TI procedure with four premises. After being exposed to all premises, the pigeons showed a consistent preference for B over D during the test. After overtraining C+D- alone, B was still preferred over D. However, the expected pattern of training performance (referred to as serial position effect) was distorted, whereas TI remained unaltered. The results are discussed regarding value transfer and reinforcement contingencies as possible mechanisms. We conclude that reinforcement contingencies can affect training performance without altering TI.

## Introduction

In the context of animal behavior, transitive inference (TI) refers to the establishment of a relation between pairs of stimuli which have not been previously trained. In the general procedure, the subject is exposed to pairs of stimuli named: A+B-, B+C-, C+D-, D+E- in a training phase, where letters represent stimuli, +means that the stimulus is followed by the delivery of a reinforcer, and - means that the stimulus was not followed by a reinforcer. After reaching over chance level of correct responses (usually more than 80% or 85%) on each pair of stimuli, the subject is exposed to non-adjacent pairs (BD, AC, AD, CE, BE, and AE) under extinction or non-differential reinforcement, which is called the test phase. The BD pair is regarded as the crucial pair, so that when the subject prefers B over D, TI is assumed. BD is regarded as the crucial pair of the so-called anchor effect. Because B and D were equally reinforced and non-reinforced, it is assumed that for solving BD TI is required.

Performance during training phase usually follows a U-shaped pattern with better performance in more extreme premise pairs (e.g., AB and DE), an effect referred to as the *serial position effect* (SPE). During the test phase, performance follows a similar pattern in which accuracy is better for more extreme premise pairs (e.g., AE) than for more central premise pairs (e.g., BD). Additionally, latencies tend to be greater for more central premises than for more extreme pairs. This pattern of performance and latencies is referred as *symbolic distance effect* (SDE). Both effects are usually reported in TI procedures (Vasconcelos, 2008).

The phenomenon of TI has been reported in different species, such as rodents (Roberts and Phelps, 1994; Takahashi and Ushitani, 2008), birds (von Fersen et al., 1991; Weiß et al., 2010; Lazareva and Wasserman, 2012; Mikolasch et al., 2013), fishes (Grosenick et al., 2007), chimpanzees (Gillan, 1981), and humans (Lazareva and Wasserman, 2010). TI has also been simulated with neural networks (Frank et al., 2003).

Different approaches have been addressed in order to explain the preference for B over D when non-adjacent pairs of stimuli are presented across test phase. According to biological approaches TI could be adaptive under conditions of social complexity where ranking possible adversaries is necessary during mating competition. Several studies with mongoose lemurs (*Eulemur mongoz*) (Maclean et al., 2008), pinyon jays (*Gymnorhinus cyanocephalus*) and scrub-jays (*Aphelocoma Californica*) (Bond et al., 2003) support this explanation, where social complexity predicts the ability to establish TI. Other studies with fishes (*A. burtoni*) (Grosenick et al., 2007) and chickens (*Gallus gallus domesticus*) (Daisley et al., 2010) indirectly support the effect of social complexity on TI.

From a reinforcement-based approach, the preference for B over D during a test cannot be predicted from the direct values based on reinforcement strength acquired during training, since both stimuli were equally reinforced and not reinforced. Therefore, in order to explain TI, it is necessary to assume that the value acquired by a particular stimulus does not depend exclusively on the number of reinforcers directly received, but it could also depend on the value transferred from other stimuli presented during each trial. This is captured by the value transfer hypothesis (Zentall and Sherburne, 1994). According to this hypothesis, B would receive more indirect value from A than the indirect value received by D from C. Because B is paired with A, which is always reinforced (A+B-), whereas D is paired with a partially reinforced C (B+C-, C+D-) and a never reinforced stimulus (D+E-). Therefore, this difference will favor more associative strength for B compared with D. Since value transfer has been shown in simultaneous discriminations (see Zentall and Sherburne, 1994) and in TI procedures (Steirn et al., 1995), the aforementioned inequity in reinforcement has been proposed as an explanation for TI. Several models based on reinforcement and value transfer hypothesis have been developed in order to explain TI (see, for example, Wynne, 1995; Siemann and Delius, 1998).

Finally, cognitive approaches appeal to mechanisms such as verbal and spatial representations, memory and logical rules. Based on this approach, TI is allowed by a ranking of the stimuli in which the most reinforced stimuli are earlier in the inferred sequence while less reinforced stimuli are later. Thus, the subject’s performance requires the storage of the serial position of each stimulus, and deficits in stablishing TI can be predicted by learning impairments or alterations in the serial position of the stimuli. Studies in animals have shown that circular arrangements of the stimuli impair TI in rats (Roberts and Phelps, 1994), which supports the idea of serial representation. Other studies have shown that hippocampal lesions may impair TI in rodents (Dusek and Eichenbaum, 1997; Heckers et al., 2004), as well as in other species such as pigeons (Strasser et al., 2004; Lazareva et al., 2015), which supports the involvement of memory in TI.

The present study is focused on the relationship between reinforcement contingencies and the formation of TI. More specifically, our aim is to explore the effect of extended training of all premise pairs and overtraining in a single premise pair. Previous studies have analyzed the effect of overtraining on TI. For example, Lazareva et al. (2004) and Lazareva and Wasserman (2006, 2012) explored the effect of overtraining the pair D+E-, as a way to increase associative strength for D, and the preference for D over B on the later test performance. In our study, we overtrained the pair C+D- (usually the most difficult discrimination to learn). Assuming value transfer, the overtraining of premise C+D- should have an indirect effect over the performance in premise B+C-. The latter premise should become more difficult to solve, because C gets more associative value through overtraining and, therefore, competes with premise B+ to receive the response. If only the direct associative strength is responsible for TI, then the effect of overtraining premise C+D- should be only a better discrimination in this pair without affecting the pigeon’s performance in the B+C- pair. Subsequent performance during the test would be disrupted.

## Materials and Methods

### Participants

Ten pigeons (*Columba livia*) maintained at approximately 85% of their free-feeding body weight by food deprivation, with permanent access to water, served as subjects. All the subjects had previous experience in an autoshaping procedure; a blue light operated as the cue. After this training, they were exposed to different ratio schedules; a white light operated as the cue. All pigeons were housed in metal cages, two pigeons were individually housed (25 cm × 25 cm × 30 cm) and eight were paired housed (40 cm × 40 cm × 45 cm). Pigeons were exposed to a 12:12 day–night cycle during the experiment, with lights on from 07:00 to 19:00 h.

The experimental procedure was approved by the local Ethical Committee of the Center for Studies and Investigations in Behavior, by the University of Guadalajara committee for animal experiments, and met governmental guidelines.

### Apparatus

Two acoustically isolated operant chambers (MED ENV-007, 25.4 cm wide × 21 cm high × 31.8 cm long). The frontal panel of the cages was flat and composed of three subpanels. The middle panel had a food hopper (ENV-123AM). Over the food hopper a 2.5 cm diameter response white-lighted cue was installed (ENV-123AM), 20.5 cm above the floor grid bars. On each side subpanel, at the same height as the white-lighted cue, it was installed a 2.5 cm diameter key that could be illuminated in different colors (ENV-131M). A house light (ENV-215M) was installed in the rear panel. Each experimental cage was placed inside a sound isolated chamber (ENV-018V) equipped with a fan (VF80A11- AC 115 v). MED-PC IV software was used for programming and recording data.

### Procedure

Pigeons were trained in five simple overlapped item pairs A+B-, B+C-, C+D-, D+E- where the positive stimuli were always reinforced and the negative stimuli were never reinforced. Stimuli A, B, C, D, and E were red, green, blue, yellow, and white cues, respectively, for all pigeons. The position of the stimuli in each trial was randomized.

The general procedure was based on the work of Lazareva and Wasserman (2010), with the main differences being the presence of correction trials, the manipulation of response rates, and the presence of correct responses criterion from one phase to other (see **Table 1**). We omitted those features trying to isolate the effect of extended exposure and pavlovian contingencies from the effect of response cost. All sessions ended after reaching the number of trials programmed for the session, or after a duration of 1 h (see **Table 1**), whichever came first.

**Table 1:** Experimental design.

	Training (A)				Test 1 (B)	Overtraining (C)		Test 2 (B)
	Phase 1	Phase 2	Phase 3	Phase 4		Phase 5	Phase 6	
Stimuli					B D			B D
			C+ D-	A+ B-	A C	C+ D-	A+ B-	A C
	A+ B-	A+ B-		B+ C-	A D		B+ C-	A D
		B+ C-		C+ D-	C E		C+ D-	C E
				D+ E-	B E		D+ E-	B E
					A E			A E
Sessions	2	2	2	9	4	1	2	1
Trials by session	200	200	200	240	200	200	200	240

*Training was divided in four phases and overtraining in two phases. Pairs were sequentially trained or overtrained following the Lazareva and Wasserman (2010) sequence. After training and overtraining, the test session of the non-adjacent pairs was also included. The number of sessions of each phase is described, as well as the maximum number of trials presented by each session. The number of pairs trained or tested by session was equally distributed*.

We used an ABCB design, where A means training, C represents overtraining and B means test (see **Table 1**).

Following Lazareva and Wasserman (2010), training (A) was divided in four phases. In phase 1 the pair A+B- was trained for 2 sessions up to 200 trials per session. Therefore, 2 more sessions (phase 2) were programmed to train the pairs A+B- and B+C-. Once again, the maximum number of trials by session was 200. Then, the pair C+D- was trained alone (phase 3) for 2 more up to 200-trials sessions each one. Finally, the four pairs were trained together (phase 4) in 9 more up to 200-trials sessions until reaching 80% of correct responses on average. In phases 2 and 4 the order of the pairs training was randomized. Therefore, at the end of training (A) each pigeon was exposed to up to 3,360 trials, distributed as follows: pair A+B- up to 1,140 trials, pair B+C- up to 740 trials, pair C+D- up to 940 trials, and finally pair D+E- up to 540 trials.

Overtraining consisted in exposition to 200 programmed trials of pair C+D- alone. Thereafter, 400 programmed trials of all premises. Therefore, overtraining consisted in a maximum amount of 600 trials for each pigeon. Considering training and overtraining, each pigeon was exposed to the following number of pairs presentations: A+B- up to 1,240 trials, pair B+C- up to 840 trials, pair C+D- up to 1240 trials, and finally pair D+E- up to 640 trials.

The day after finishing training and overtraining phases one test session was programmed. In each test session, up to 240 trials of non-adjacent pairs BD, AC, AD, CE, BE, and AE were presented in random order under non-differential reinforcement.

A trial started with the house light and white center key illuminated. After the white key was pecked, it turned off and the side keys were illuminated. The first response in one of the two side keys turned them off, and a reinforcer was delivered or not depending on the chosen stimulus.

During training and overtraining, correct choices (i.e., reinforced stimuli in the pair presented: +) were always followed by 4 s of food access to the food hopper that operated as the reinforcer. After a correct choice, an inter-trial interval (ITI) of 10 s was inserted. After an incorrect choice (i.e., non-reinforced stimuli in the pair presented: -), no reinforcer was delivered and the ITI lasted 14 s. During the ITIs the house light stayed off.

During tests, a non-differential reinforcement schedule operated. Thus, both premises on each pair were partially reinforced with an equal probability of 0.5.

### Results

To explore the effect of training and overtraining, we considered the average percentage of correct choices and latencies of response on each premise for the last two sessions of training (i.e., Phase 4) and overtraining (i.e., Phase 6). For test phases we considered only the last two sessions of Test 1 and the single session of Test 2. Because we found in a few rare trials very extreme latencies, especially during test phases, we did not consider for analysis trials with latencies higher than 2 s. For all phases, latencies showed a positive asymmetrical distribution. Thus, we estimated the average of median latencies for each session considered. All statistical analyses were performed with SPSS v20.

The total number of programmed presentations of each premise was not the same (see **Table 1**). **Table 2** shows the number of trails completed for all subjects and sessions for each premise, as well as the average percentage of correct responses considering only the two last sessions of training and overtraining.

**Table 2:** Number of effective presentations of each pair regarding all subjects and sessions across training and overtraining.

Premises	# of presentations	
	Training	Overtraining
A+B-	8347 (78.72 ± 3.86)	9331 (86.68 ± 2.86)
B+C-	5902 (87.21 ± 2.05)	6888 (47.03 ± 5.57)
C+D-	7448 (40.28 ± 5.44)	10400 (78.56 ± 3.46)
D+E-	4360 (91.08 ± 1.74)	5363 (84.26 ± 3.83)

*In parenthesis appears the average percentage of correct responses considering the last two sessions of training and overtraining phases*.

**FIGURE 1:**
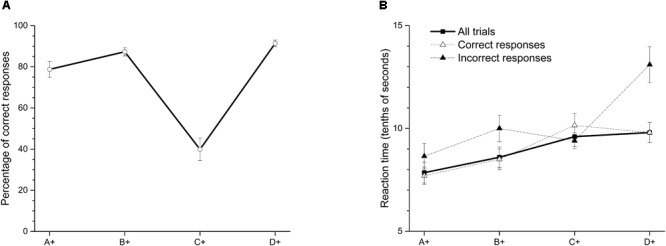
**(A)** Average percentage (±SEM) of correct responses and **(B)** reaction time (tenths of second) on each stimulus in the last two sessions of training.

**Figure 1A** shows the average percentage of correct responses in the last two sessions of training. Each dot represents the average percentage of correct responses in the pair where the stimulus was always reinforced. We found a function that resembles a SPE after training, with high percentages of correct responses in more extreme premises.

Our data show that, as has been previously reported, the worst performance is in premise C+D- (39.87%), and high accuracies are in the most extreme premises, D+E- (91.36%) and A+B- (78.76%). A within-subjects ANOVA with Greenhouse-Geisser correction showed differences in accuracy between premises [*F*(2.03,18.27) = 49.89; *p* < 0.01]. The Bonferroni *post hoc* test showed that accuracy in premise C+D- differed from the other three premises (*p* < 0.01), and the accuracy in premise A+B- was statistically higher than in premise C+D- (*p* < 0.05) and premise D+E- (*p* < 0.05). B+C- did not differ from D+E- (*p* > 0.05). Additionally, a one-sample *t*-test revealed that accuracy in premise C+D- did not differ from chance [*t*(9) = -1.97; *p*= 0.08]. Latencies increased as a function of serial position (see **Figure 1B**), meaning that they were shorter for the A+B- pair than for the D+E- pair. Significant differences were found between premises [*F*(2.16,19.4) = 15.73; *p* < 0.01] using a within-subjects ANOVA with Greenhouse-Geisser correction. Latencies in premise A+B- were shorter than in premises C+D- and D+E-, but they were apparently different from the B+C- latency. Premise A+B- is the only pair in which one stimulus (A+) is always reinforced across the training. In contrast, latency in premise D+E-, being the only pair in which one stimulus (E-) is never reinforced, was the highest, but it did not differ of latencies corresponding to premises B+C- and C+D-.

**FIGURE 2:**
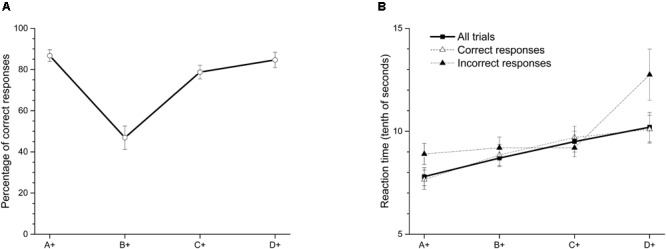
**(A)** Average percentage (±SEM) of correct responses and **(B)** reaction time (tenths of second) on each stimulus in the last two sessions of overtraining premise C+D-.

As an attempt to correct deficits in premise C+D- after training phase, we increased training in pair C+D- during overtraining phase (see **Table 2**), so that at the end this pair was the premise most trained across sessions. As far as we know, this manipulation has not been done before. After overtraining this premise (see **Figure 2A**), the average accuracy in C+D- increased to 78.74% (*SEM* = 3.32), whereas performance on B+C- significantly decreased to 46.9% (*SEM* = 5.71). A within-subject ANOVA with Greenhouse-Geisser correction showed differences in accuracy between premises [*F*(2.29,20.06) = 25.44; *p* < 0.01]. The Bonferroni *post hoc* test showed that only the accuracy in premise B+C- differed from the other three premises (*p* < 0.05). No further differences were observed between premises. The accuracy in premise B+C- did not differ from chance [*t*(9) = -0.57; *p* = 0.58]. Latencies for each premise (see **Figure 2B**) stayed as in previous training. Once again, latency increased as a function of serial position.

A change in performance from Test 1 to Test 2 would be expected after overtraining the premise C+D-, regarding exclusively the frequency of reinforcement for each premise (see **Table 2**). Nevertheless, because performance in both Tests was equal, we will describe both tests at the same time.

Our data were not consistent with any hypothesis of TI based on the frequency of reinforcement of each premise. There was no effect of overtraining premise C+D- on test performance. We observed an increase in accuracy as a function of symbolic distance between stimuli.

**FIGURE 3:**
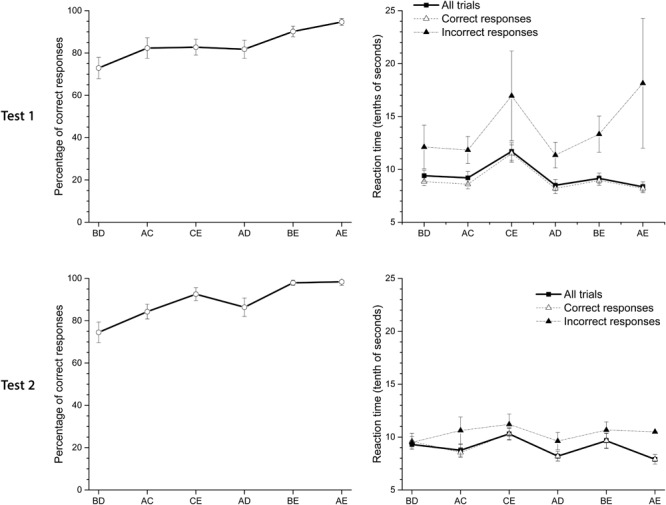
Average percentage (±SEM) of correct responses and reaction time (tenths of second) in Test 1 and Test 2. From left to right, pairs of stimuli are ordered by their symbolic distance.

**Figure 3** shows the pigeons’ performance and latencies of response on Test 1 and Test 2 when stimuli were simultaneously presented in non-adjacent pairs. The averaged pigeon performance (see **Figure 3**) is consistent with the SDE. Therefore, the worst performance is in pair BD and the best is in pair AE. During Test 1 the averaged accuracy in the most central pair, BD, differed significantly from the most distal pair, AE, and pair BE [*F*(2.28,20.5) = 0.67; *p* < 0.01]. The same pattern was observed in Test 2. Pigeons also showed a slight and non-significant decrease in latencies as symbolic distance increased. Latency in pair CE was significantly higher than in the other pairs. Exactly the same pattern was observed in Test 2, with a slightly better performance in accuracy and less variable latencies. Latencies for incorrect responses were higher than for correct responses. Considering all the premise pairs, latencies slightly decreased as the distance between non-adjacent premises on each pair increased.

Therefore, TI was established in the absence of correction trials and despite deficits in performance during premise C+D. This pattern of performance during the test remained even when overtraining was administered. The preference for B stimulus in pair BD, was higher and differed from chance for Test 1 [*t*(9) = 4.83; *p* < 0.01] and Test 2 [*t*(9) = 4.48; *p* < 0.01].

## Discussion

Our data show that using the employed training, pigeons were able to establish TI in the absence of correction trials and even when performance deficits in C+D- were found and persisted across the Training Phase. Overtraining the premise pair C+D- did not affect TI but distorted SPE, so that three pairs (i.e., A+B-, C+D-, D+E-) reached the highest accuracy and only one pair (B+C-) dropped near to chance. However, this extra training in C+D- did not affect TI. The performance in the worst solved premise pair before overtraining (i.e., C+D-) and after overtraining (i.e., B+C-) did not differ from chance.

Latencies increased after training related to SPE. Therefore, for premise A the latency was shorter than for premise B, and so on. Exactly the same was observed after overtraining. In both tests (after training and overtraining), latencies slightly decreased as the symbolic distance between non-adjacent premise pairs increased. The higher latency was observed in the pair CE. Interestingly, latencies were always shorter for correct responses, no matter what the non-adjacent premise pair presented, than for incorrect responses, except for the most inner pair BD.

Because we did not counterbalance the stimuli’s color between pigeons, it is possible to argue that this could affect the choices. Besides, counterbalancing colors is a common practice in TI procedures. However, some previous studies have shown that reversing the order of colors and pre-exposition to the same colors during training do not affect TI performance. For example, Strasser et al. (2004) used figures as stimuli but without counterbalancing and they found TI. Additionally, Steirn et al. (1995) conducted two experiments with relevance to this question. During the first one, they assigned one group to a set of premises A+B-… D+E- (group A+) and the other to the reverse A-B+… D-E+ (group A-). All pigeons had previous exposition to the same stimuli employed during training. They only found more variability in the amount of sessions to reach the criterion (90% of correct responses during two consecutive sessions) for group A- with no statistical differences during test pair BD. Moreover, in their second experiment, they compared pigeons with previous experience with the stimuli and naïve pigeons, finding no differences to reach the criterion, although there was a lower performance in naïve pigeons during the first sessions of each phase. The performance during the test did not differ between groups. Therefore, it seems that previous experience with the stimuli and the counterbalance of stimuli can slightly affect performance, however, TI seems to be unaffected.

Because overtraining premise pair C+D- seems to not have an effect over TI, but it decreased the discrimination of pair B+C- during overtraining, we will discuss first the effects of training and overtraining over premise pairs discrimination and later, we will discuss its effect on TI. Finally, we will focus on latencies of response during training (and overtraining) and tests.

### Training and Overtraining Effects on Premise Pairs Discrimination

Vasconcelos (2008) describes the U-shape as the idealized curve performance for the five-term series. However, the shape of the curve can take several forms depending on many features during training. There are many ways for training the premise pairs and consequently different outcomes in acquisition. Studies differ in the amount of training trials. For example, von Fersen et al. (1991) trained the complete set of stimuli across all sessions reaching 27,000 stimulus pair presentations, and they found the expected U-shape in performance with naïve pigeons as subjects. Steirn et al. (1995) reported above chance performance and TI with only 2,112 training trials. Siemann et al. (1996) reported 2,700 trials to reach above chance performance and TI, and their performance curve resembles an exponential ascending function. Therefore, there is not a specific criterion that determines the number of trials or sessions necessary to establish above chance performance and TI, and the idealized U-shape is not always found.

After our training procedure, the expected U-shape was not exactly found, although performance above chance was reached for all premise pairs except C+D-. This finding does not resemble precisely what other studies have found. For example, in von Fersen et al. (1991) the pair C+D- is the worst solved but it is still above chance. In Siemann et al. (1996), the pigeons’ performance is noticeable better, since they found an ascending function from A+ to D+ (contrary to the expected U-shape), but the training began with central premise pairs (B+C- and C+D-) and then proceeded with extreme pairs, first pair A+B- followed by pair D+E-. They used a different number of presentations of each pair and imposed a criterion of 80% of correct responses before starting the test phase to avoid the emergence of an end-anchor effect. Other studies have also found this ascending function in performance. For example, Lazareva and Wasserman (2006) -with pigeons- and Lazareva and Wasserman (2010) -with humans- also reported ascending training performances from A+B- to D+E- (from 80 to 90%) using the same pattern of premise training as we did. In this respect, it is important to point out that in both studies manipulations in response rates and correction trials were employed, which could have improved accuracy for all premise pairs, whereas in our experiment the absence of those manipulations could have provoked deficits in performance. Support for this hypothesis comes from serial learning studies. For example, Straub and Terrace (1981) employing pigeons as subjects, trained a sequence of stimuli A→B→C→D where “→” denotes the consequent stimulus and the pecking of the last stimulus in the chain delivered the reinforcer. With gradual training (A→B, A→B→C and A→B→C→D) and different levels of correct responses required (25, 50, and 70%), the performance showed different shapes, having “C” the worst performance with the complete chain trained at the lowest correct responses requirement. They found a performance curve that closely resembles the one we found, which suggests that in absence of correction trials and with lower correct responses requirement, the learning of relations between stimuli is impaired by the addition of a third stimulus. Nevertheless, the fact that C+D- was constantly the worst solved across four sessions of training all premises, cannot be explained by this assumption.

It is also worth mentioning that other studies do not report the U-shape during training although TI is still reported (see Strasser et al., 2004 with pigeons, and Acuna et al., 2002 with humans). Therefore, the observed differences in performance depending on training administration, and especially the absence of the U-shape, deserve further verification.

**FIGURE 4:**
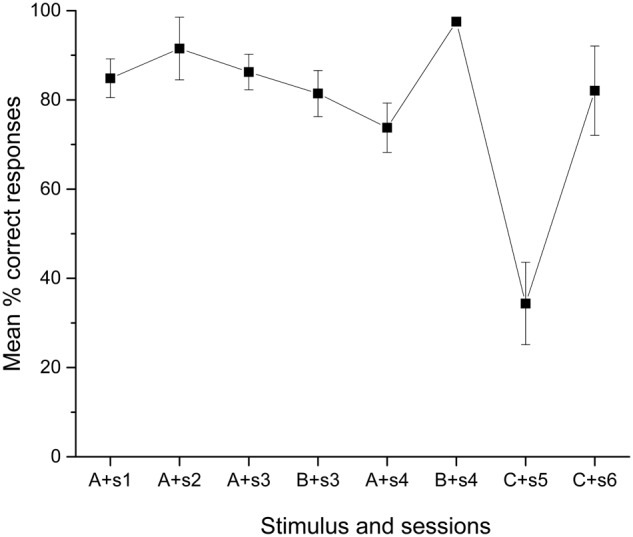
Mean percentage (SEM±) of correct responses for each stimulus until Phase 3 (sessions 1 to 6).

There is evidence in humans in which learning a second list of verbal stimuli decreases the remembered items for the first list, an effect referred to as retroactive inhibition (Barnes and Underwood, 1959). If this phenomenon affected extensively trained stimuli, the observed effect of overtraining C+D- could be explained. In fact, looking at the performance during the first training session in the pair C+D-, the percentage of correct responses was below chance but without significance [34.36%; *t*(9) = -1.691, *p* = 0.13], which did not happen with any other pair, but significantly improved over chance [82.06%; *t*(9) = 3.201, *p* = 0.01] by the second training session (see **Figure 4**).

**FIGURE 5:**
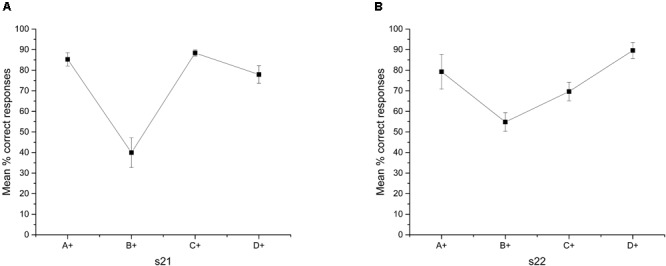
Mean percentage (SEM±) of correct responses for each stimulus during overtraining phase. **(A)** Session 21 and **(B)** Session 22.

However, the performance in C+D-, never over chance, remained stable across the entire Phase 4 (data not shown), and improves during overtraining. Therefore, it is unclear why the discrimination C+D- is correctly solved when presented alone but poorly solved when presented along with the other premise pairs. Additionally, the improvement in C+D- diminishes from the first to the second session after overtraining (see **Figure 5**), so that the function acquires a U-shape with B+C- being the worst pair solved and D+E- the best solved.

More direct evidence related to overtraining comes from studies in TI where bias reversal is employed. For example, in Lazareva et al. (2004), after training all premise pairs, their subjects -hooded crows- were exposed to D+E- presentations until reaching a reinforcement ratio in D greater than in B. Despite of this manipulation, B was preferred over D (especially in the ordered feedback group). Lazareva and Wasserman (2006), using pigeons, employed the same bias reversal and found a slight improvement in general performance during training, but the preference of B over D remained the same during the test. The same effect of bias reversal has been found in Lazareva and Wasserman (2012) using pigeons. Nevertheless, in a more recent study, Lazareva et al. (2015) have shown that bias reversal is reported (changing BD preference) in some pigeons but not in others, which suggests that in some pigeons, performance depends on reinforcement history, whereas in others, performance depends on the implicit order of the stimuli.

In the present experiment, overtraining C+D- unexpectedly decreased performance in B+C- discrimination. However, that decrement did not change BD preference in any test. It is worth mentioning that in all the previously mentioned studies, bias reversal on D+E- slightly improved general performance during training, whereas in our procedure overtraining C+D- distorted general performance. This fact suggests that methodological differences with others experiments, such as using correction trials or the response requirement, have noticeable effects on the expected U-shape during training.

### Tests Performance

In our results, despite the already mentioned deficits during performance, particularly for C+D-, and despite the changes provoked by the extra training in C+D- (which lowered B+C- near to chance), the preference for B over D and the complete pattern of performance during Tests 1 and 2 remained unaffected. This finding turns out to be unexpected based on the mere calculations of reinforcement ratios, and it suggests that learning during acquisition and performance during test could be affected by independent mechanisms. It would then follow that impairments during training do not always translate into impairments during test. Siemann et al. (1996) pointed out a similar argument, stating that with sufficient training reinforcement histories have minor consequences in TI. Steirn et al. (1995) reported a negative correlation between reinforced responses during B+C- and D+E- and the preference of B over D. Moreover, they found a preference of B over D even when the premise pairs’ training (A+B-, C-E+, C+D-, A+E-) did not support it by reinforcement history. This trend has also been shown in monkeys, where TI was established despite predictions from reinforcement ratios (Boysen et al., 1993). More recently, Daniels et al. (2014) did not find a significant correlation between the proportion of relative reinforcement and TI performance in pigeons.

There are also indirect evidence supporting this independency between training and test performance. For example, Strasser et al. (2004) did not find differences during training and TI between hippocampal-lesioned and intact pigeons, which would imply that impaired spatial representation (caused by hippocampal lesions) does not affect neither acquisition nor TI. Similar findings can also be found in reinforcement models. For example, Lazareva et al. (2004) using crows (*Corvus cornix* L.) as subjects reported TI, but when they applied two different models of associative strength these models successfully fit the data from training but failed to fit BD performance. In another study from Lazareva and Wasserman (2006), both associative models failed to predict BD performance in pigeons after bias reversal. A similar inconsistency can also be found in humans (Lazareva and Wasserman, 2010) and pigeons (see Lazareva and Wasserman, 2012), where associative models produced better prediction for training than for testing.

Looking at the total reinforcement ratios after the training of all premise pairs and the over-trained premise pair C+D- (see **Table 2**), it seems that reinforcement ratios cannot account for the persistence of TI in our results, since B did not have a greater value than D. Performance during training and its effect on the tests seem unable to be predicted by inequalities of reinforcement.

On the other hand, cognitive approaches would be also insufficient, since the extended exposure to all premise pairs (Phase 4 of training) did not improve C+D- performance (data not shown). Additionally, overtraining the pair C+D- impaired B+C- performance without affecting TI. Studies in humans have shown that extended training can improve working memory even in long-term periods and with transference to different tasks (see Klingberg, 2010 for a review). However, the effect of extended training in TI procedures remain elusive. Studies with neural networks have found that overtraining specific premise pairs (for example, pair E+F- in a seven-term series) in impaired networks improves performance during BD pair (Frank et al., 2003) but without reporting effects during training, which turns out to be inconsistent with our findings.

Considering that the direct values of reinforcement cannot explain the observed pattern, we explore the possible effect of value transfer. In order to calculate values in indirect associative strength, we took the formula of value transfer theory (VTT) from von Fersen et al. (1991): V _i_ = R_i_ + a^∗^V _i+1_, where *V*_i_ represents indirect value of a particular stimulus when it is paired with a reinforced stimulus, *R*_i_ is the value from the stimulus directly reinforced, *a* is a weighting parameter (ranging from 0.1 to 0.5), and *V*_i+1_ is the value of the rewarded stimulus when presented with stimulus *i*.

Taking the overall performance, we used the reinforcement ratios [rewarded trails/(rewarded + non-rewarded trials)] and updated the values from session 1 to session 15 based on parameter *a*. Then, the last two training sessions before overtraining were averaged and contrasted with the obtained data. Taking those values, our data do not support value transfer, since running the equation the best fitting obtained with least squares method was with *a* near to zero (data not showed), which would imply no value transfer. Despite of several models which assume value transfer (Wynne, 1995; Siemann and Delius, 1998), other studies have also suggested TI in absence of value transfer (Weaver et al., 1997). Regarding the above mentioned, although value transfer remains as a possible intervening mechanism in TI procedures our data neither support an explanation based on value transfer.

### Latencies

Latencies during performance remained as an ascending pattern from the most central to the most extreme premises, which turns out to be contrary to the widely reported SDE. This finding is also inconsistent with the decrement in latencies predicted by cognitive approaches as an effect of practice (Logan, 1992). In a previous study we found a monotonic decrement in latencies as a function of extended exposure to two alternatives predicted by a power law (Camarena-Pérez and García-Leal, 2017). This effect has been also reported in perceptual discrimination procedures in monkeys, where more alternatives provoke less steep decrease in latencies (Albantakis and Deco, 2009). Whether a comparison process between alternatives were operating, larger latencies would be expected in pair BD and shorter latencies in the pair AE, contrary to our findings. On the other hand, if latencies decrease as a function of practice, we would have found steeper or flat functions instead of ascending latency function.

How latencies change in TI procedures seems to be more widely reported in humans (Woocher et al., 1978; Acuna et al., 2002; Munelly et al., 2010) than in non-human animals. D’Amato and Colombo (1990) reported increasing latencies in monkeys (*Cebus paella*) during testing when they grouped pairs containing A, B, C, and D as the first stimulus in the pair. The same trend is shown in D’Amato and Colombo (1988). These findings are regarded by them as SDE.

The valuation process (if any) in TI procedures remains unclear, and along with the fact that not all TI studies reported data about latencies, the question as to what should be the observed trend in latencies remains unanswered. Previous studies about the effects of practice in learning curves have suggested a hyperbolic improvement until reaching an asymptotic performance across sessions, as an accumulation of correct responses (Thurstone, 1919; Mazur and Hastie, 1978). For the present experiment, that hyperbolic improvement is clearly absent, which in turn suggests that learning of TI cannot be explained by mere accumulation of correct responses.

## Conclusion

TI can be established even with deficits in performance during acquisition and overtraining in one premise. Nevertheless, it remains unclear why the overtraining of one single premise did not affect TI and impaired performance of the immediate previous premise. Our findings suggest that the specific way in which premises are trained could have effects on the expected functions of performance without affecting TI, and that even when subjects can discriminate a single premise alone, this discrimination becomes impaired when all premises are presented together. Studies focusing on the performance premise by premise and session by session, along with a more detailed analysis of latencies, could be helpful in determining the specific mechanisms involved in the establishment of TI.

## Author Contributions

All authors jointly designed the experiment and discussed the results. HC collected the data. HC and OG-L jointly wrote the manuscript.

## Conflict of Interest Statement

The authors declare that the research was conducted in the absence of any commercial or financial relationships that could be construed as a potential conflict of interest.
