# Corrigendum: Transitive Inference Remains Despite Overtraining on Premise Pair C+D–

**DOI:** 10.3389/fpsyg.2019.00099

**Published:** 2019-01-30

**Authors:** Héctor O. Camarena, Oscar García-Leal, José E. Burgos, Felipe Parrado, Laurent Ávila-Chauvet

**Affiliations:** Center for Studies and Investigations in Behavior, University of Guadalajara, Guadalajara, Mexico

**Keywords:** transitive inference, value transfer, reinforcement, overtraining, bias reversal

In the original article, there was an error. The letter “C” was incorrectly provided and should instead be “B.”

A correction has been made to the **Introduction**, paragraph seven:

“The present study is focused on the relationship between reinforcement contingencies and the formation of TI. More specifically, our aim is to explore the effect of extended training of all premise pairs and overtraining in a single premise pair. Previous studies have analyzed the effect of overtraining on TI. For example, Lazareva et al. (2004) and Lazareva and Wasserman (2006, 2012) explored the effect of overtraining the pair D+E−, as a way to increase associative strength for D, and the preference for D over B on the later test performance. In our study, we overtrained the pair C+D− (usually the most difficult discrimination to learn). Assuming value transfer, the overtraining of premise C+D− should have an indirect effect over the performance in premise B+C−. The latter premise should become more difficult to solve, because C gets more associative value through overtraining and, therefore, competes with premise B+ to receive the response. If only the direct associative strength is responsible for TI, then the effect of overtraining premise C+D− should be only a better discrimination in this pair without affecting the pigeon's performance in the B+C− pair. Subsequent performance during the test would be disrupted.”

The authors apologize for this error and state that this does not change the scientific conclusions of the article in any way. The original article has been updated.

## Conflict of Interest Statement

The authors declare that the research was conducted in the absence of any commercial or financial relationships that could be construed as a potential conflict of interest.
